# How social–emotional learning promotes reading achievement? A systematic review of mechanisms and instructional design

**DOI:** 10.3389/fpsyg.2025.1631429

**Published:** 2025-12-30

**Authors:** Li Hua, Zhenlin Zhang, Honglan Wang

**Affiliations:** 1 School of Physics and Electronic Information Engineering, Hubei Engineering University, Xiaogan, China; 2 School of Education and Psychology, Hubei Engineering University, Xiaogan, China

**Keywords:** children, primary school, reading achievement, social-emotional learning, teaching strategy

## Abstract

**Introduction:**

Social-Emotional Learning (SEL) has garnered significant attention within the field of education due to its potential to promote the holistic development of students. While a substantial body of research substantiates the role of SEL in enhancing social and emotional growth, there remains a relative paucity of knowledge regarding its specific effects on reading achievement. This review addresses two pivotal questions: (1) How does SEL specifically influence students’ reading achievement? and (2) How can teachers design and implement SEL-integrated instructional materials to enhance reading achievement?

**Methods:**

A systematic literature review was conducted in accordance with PRISMA guidelines. Thirty-one peer-reviewed empirical studies published up to August 2024 were identified from major databases, including Web of Science and Scopus. The inclusion criteria focused on studies examining both social-emotional learning and reading achievement among school-aged learners.

**Results:**

The synthesis of findings indicates that SEL enhances reading achievement through four interrelated pathways: improving emotional regulation, increasing intrinsic motivation, strengthening peer and teacher–student interactions, and fostering long-term reading behaviors. In addition, SEL is associated with improvements in reading comprehension, vocabulary acquisition, and metacognitive awareness.

**Discussion:**

Overall, the evidence underscores the critical role of SEL as a foundational component of reading instruction rather than a supplementary approach. The findings offer meaningful implications for teacher training, curriculum design, and educational policy aimed at improving students’ reading achievement through SEL-integrated instructional practices.

## 1 Introduction

In recent years, Social and Emotional Learning (SEL) has emerged as a focal point in educational discourse, showing important role in fostering students’ holistic development. SEL encompasses the process through which individuals develop crucial skills in emotional regulation, relationship development, and responsible decision-making (CASEL, 2020). Numerous studies have established a positive association between the social–emotional skills of SEL program participants and their academic performance (Wilson et al., 2001; Fernández-Martín et al., 2021). As Durlak et al. (2011) emphasized, “the 11-percentile gain in academic performance achieved in these programs is noteworthy, especially for educational policy and practice” (p. 417) Notably, several researchers highlighted SEL’s specific efficacy in supporting reading achievement (Jones et al., 2011; Mahoney et al., 2018). Vygotsky’s (1978) sociocultural theory emphasizes that the social interaction is integral to cognitive development, providing a theoretical basis through which the impact of SEL on reading can be understood. Yu et al. (2023) found that emotional awareness and empathy significantly enhanced reading achievement, particularly in peer-supported learning environments. Zimmerman’s (2002) self-regulated learning model underscored the importance of emotional control in fostering reading achievement, while Ross and Tolan (2018) demonstrated that emotional strategies contributed to improving reading fluency.

Drawing upon Immordino-Yang’s (2016) affective-cognitive framework, which posited that reading was shaped by emotional engagement and cognitive regulation, empirical studies have illuminated the importance of SEL in facilitating reading achievement. Emotional involvement significantly enhances memory consolidation and attention focus during reading tasks (Zaccoletti et al., 2020), while SEL competencies promote positive behaviors and peer interactions, both of which have been shown to contribute substantially to improving reading achievement (McKown et al., 2016). SEL also plays a pivotal role in the development of metacognitive skills like self-monitoring and reflection thinking (Durlak et al., 2011). Furthermore, the emotional regulation component of SEL effectively mitigates anxiety-related obstacles to reading (Zaccoletti et al., 2020).

Despite the growth of empirical research on SEL, most studies emphasize its overall benefits without disentangling the unique contributions of individual components. However, emerging evidence indicates that specific SEL skills including empathy, emotional regulation, and social comprehension may impact reading achievement in indirect ways. These effects are typically mediated by behavioral regulation or social adjustment rather than direct causality (Wanless and Domitrovich, 2015; McKown et al., 2016; Ross and Tolan, 2018; Chen and Yu, 2022). Such complexities underscore the pressing need to investigate how SEL specifically shapes reading achievement, particularly within multilingual educational settings. This imperative has been echoed by international policy-making bodies. UNESCO (2022) highlighted the critical importance of integrating SEL into national curricula and teacher education programs to enhance both academic performance and socio-emotional well-being. Similarly, the OECD’s, 2024 Global Scoping Study on SEL further revealed that only 21% of 15-year-old students attended schools where both principals and teachers actively support SEL initiatives. Significantly, these schools reported higher reading achievement and greater learning motivation, underscoring the urgency of incorporating SEL into reading instruction. Teachers serve as pivotal agents in turning SEL into instructional practices. Schonert-Reichl (2019) emphasized that teachers’ emotional support and effective classroom management significantly exerted influence on students’ academic motivation and engagement. Poulou and Garner (2023) further demonstrated that emotionally intelligent educators were instrumental in fostering empathy, encouraging active participation, and promoting peer collaboration in the classroom. While existing research has underscored the importance of integrating SEL into teaching strategies and academic content, aligning it with academic learning objectives, and leveraging reading activities to develop emotional skills, few studies have proposed practical, operational strategies for classroom implementation (Jennings and Greenberg, 2009; Herrenkohl et al., 2019; Ng and Sun, 2022).

To bridge these critical gaps, the current study undertakes an in-depth exploration of two practice-oriented research questions: (1) How does SEL specifically influence students’ reading achievement? and (2) How can teachers design and implement SEL-integrated instructional materials to foster reading achievement? The findings are expected to have far-reaching implications. For educators, a more profound comprehension of how SEL specifically impacts reading achievement will enable them to refine their instructional strategies. This understanding serves as a foundation for developing more effective instructional approaches and facilitating the wider implementation of SEL programs focused on improving reading proficiency. For students, the insights will turn into more effective SEL interventions. Educational administrators stand to benefit as well. The results inform the implementation of policies that are rooted in new information, thereby leveraging reading improvement initiatives. For researchers, this study paves the way for future exploration and practical application.

The subsequent sections of this paper will provide a comprehensive exploration of the study. The second section offers an in-depth account of the research methodology about how to get the literature related to Social–Emotional Learning (SEL) and reading achievement. The third section presents the research findings and discusses their implications. The fourth section concludes with recommendations for future research and educational practice.

Previous studies have employed various terms, such as reading performance, reading outcomes, or reading efficiency to describe literacy-related results. Reading efficiency often emphasizes processing speed or fluency rather than comprehensive academic outcomes. In this review, we consistently adopt the term reading achievement to refer to standardized or curriculum-aligned reading outcomes while noting original authors’ terminology when relevant.

## 2 Method

### 2.1 Search strategy

This paper is based on a comprehensive literature search aimed at identifying empirical studies that explore the impact of SEL on children’s reading achievement. Two prominent academic databases Web of Science and Scopus were systematically accessed to retrieve relevant studies published until August 2024. The search strategy employed Boolean operators (“AND,” “OR”), different expressions of SEL (Corcoran et al., 2018; Fernández-Martín et al., 2021; Blewitt et al., 2019), reading achievement (Wasik et al., 2016; Snyder et al., 2016), and child populations (Martins et al., 2022; Evangelio et al., 2022; Dudley et al., 2015). This decision was intended to avoid the false negatives likely to arise from the more generic use of the term SEL in articles. Only peer-reviewed journal articles written in English were included in the analysis. The initial query yielded 707 records from Web of Science, 943 from Scopus, and an additional 4 from Google Scholar. Following the removal of 490 duplicates, a total of 1,168 articles remained for title and abstract screening. Further details regarding the search output and the entire process can be found in the Supplementary material.

Titles and abstracts are first reviewed for relevance, followed by full texts that are retrieved and assessed to ensure alignment with the research questions and inclusion criteria. Studies are included if their titles, abstracts, or full texts: (1) directly refer to SEL and reading; and (2) employ quantitative, qualitative, or mixed-method approaches for data collection and analysis.

### 2.2 Inclusion criteria

**Figure 1:**
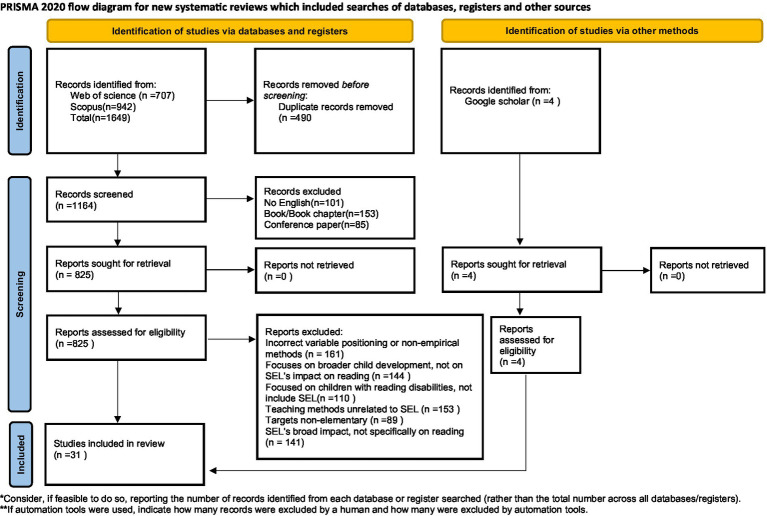
The process of article selection. Source: Page MJ, et al. BMJ 2021:372: n71. doi: 10.1136/bmj.n71.

Studies are included if SEL or reading is implicitly examined through intervention or assessment, even without explicit mention in the title or abstract. Interdisciplinary studies spanning educational psychology, child development, and reading instruction are considered eligible. Furthermore, studies authored by researchers with an established publication record in SEL or reading research are also retained, as such studies often offer in-depth theoretical insight. Non-English articles, book chapters, theses, conference proceedings, and articles lacking empirical data are excluded. Adhering to these criteria, a final sample of 31 peer-reviewed studies is selected for review. All full texts are independently reviewed and verified by the research team. The article selection process complies with the Preferred Reporting of Items for Systematic Reviews and Meta-Analyses (PRISMA) Statement (Moher et al., 2009) (Figure 1).

### 2.3 Extraction and analysis

Data from the selected articles is extracted across six key dimensions: (a) author(s), (b) year of publication, (c) geographical context, (d) study design, (e) research participants, and (f) key results or emergent themes. Thematic synthesis is then employed to identify shared mechanisms, strategies, and outcomes across studies, aligned with the two guiding research questions.

## 3 Findings

As shown in Table 1, the thirty-one reviewed studies were systematically analyzed and organized according to their dominant mechanistic pathways linking Social–Emotional Learning (SEL) to reading achievement. Rather than presenting the studies in a purely descriptive manner, this analytical framework highlights how different SEL processes operate as mediating or facilitating mechanisms for literacy development.

**Table 1:** Analysis of included studies.

Author(s)	Geographical context	Study design	Research participants	Key results / Emergent themes related to reading achievement
Emotional regulation pathway				
Pellitteri et al. (2006)	USA	Conceptual review	Students with reading disabilities	Emotional intelligence training boosted reading motivation and comprehension.
Jones et al. (2010)	USA	School RCT	942 Grade 3 students	Integrated SEL and literacy (4Rs) program raised reading scores and attendance.
Jones et al. (2011)	USA	Longitudinal RCT	1,184 students	Two-year 4Rs exposure improved reading achievement for behavioral risk students.
Ashdown and Bernard (2012)	Australia	Quantitative	99 Grade 1 students	Explicit SEL instruction enhanced students’ social–emotional skills and reading achievement.
Brackett et al. (2012)	USA	Quantitative	273 Grade 5–6 students	RULER curriculum improved reading performance and social–emotional competence.
Nix et al. (2013)	USA (PA)	RCT	356 preschoolers	Preschool SEL skills predicted later reading achievement.
Schonfeld et al. (2015)	USA (urban)	Cluster RCT	705 elementary students	PATHS intervention raised reading scores in high-risk schools.
McKown et al. (2016)	USA	Quantitative (SEM)	340 elementary students	SEL comprehension predicted social skills and reading performance.
Akhter et al. (2020)	Pakistan	Correlational	209 Grade 8 students	Emotion regulation positively associated with reading performance.
Daunic et al. (2021)	USA (SE)	Cluster RCT	1,154 K–1 students	SEL–literacy curriculum improved vocabulary, behavior, and self-regulation.
Daunic et al. (2021)	USA	RCT	1,154 students	SEL Foundations curriculum enhanced self-control and literacy skills.
Motivational and engagement pathway				
Rimm-Kaufman et al. (2007)	USA	Longitudinal	Grade 3–5 students	Responsive Classroom approach enhanced reading achievement over 3 years.
McCormick et al. (2015)	USA	Quantitative (causal methods)	Low-income K–1 students	Classroom-level SEL mediated reading achievement via engagement.
Panayiotou et al. (2019)	England	Quantitative	1,626 students	SEL skills predicted reading achievement through motivation and engagement.
Hart et al. (2020)	USA	Cluster RCT	51 Grade 2 classes	Universal SEL had positive effect on future reading test performance.
Ha and Roehrig (2022)	USA (Florida)	Quantitative	67 students	Reading motivation and prosocial efficacy jointly predicted reading growth.
Social interaction and prosocial behavior pathway				
Jennings and Greenberg (2009)	USA	Theoretical review	Teachers	Teacher SEL competence improved classroom climate and student engagement.
Denham and Brown (2010)	USA	Review	Preschool children	SEL competencies linked to self-regulation and literacy readiness.
Wanless and Domitrovich (2015)	USA	Review	Teachers	Implementation readiness affects SEL quality and literacy outcomes.
Kozak and Recchia (2018)	Canada	Qualitative review	Elementary students	Reading fiction cultivates empathy and social understanding that support literacy.
Ng and Sun (2022)	Singapore	Qualitative	19 kindergarten classes	Shared book reading facilitated interpersonal and intrapersonal SEL, improving literacy discussions and empathy.
Todd et al. (2022)	USA	Qualitative	K–12 teachers	Practitioners perceived SEL integration as enhancing reading engagement.
Cognitive and metacognitive pathway				
Durlak et al. (2011)	USA	Meta-analysis	270,034 students	Universal SEL interventions enhanced reading and academic achievement.
Corcoran et al. (2018)	International	Meta-analysis	PreK–12 (>57 k reading cases)	SEL programs improved reading achievement (ES ≈ 0.25).
Mahoney et al. (2018)	USA	Meta-review	356 studies	SEL yielded ~11 percentile gains in reading achievement.
Wang et al. (2019)	Western China	Quantitative	7,106 students	SEL competencies predicted cognitive and reading development.
Chen and Yu (2022)	China	Meta-analysis	8,736 students (86 studies)	SEL in China significantly improved SEC and academic outcomes but less on behavioral problems.
Hayashi et al. (2022)	Japan, Africa, North America	Scoping review	42 empirical studies	Proposed culturally relevant SEL model linking embodied learning and academic achievement.
Chen (2024)	China	Conceptual review	Teachers and schools	Identified implementation strategies for SEL integration in education to improve learning and well-being.

Four recurrent pathways emerged from the synthesis. The Emotional Regulation Pathway (*n* = 11) represents the largest group of studies, emphasizing how children’s ability to manage emotions, sustain attention, and regulate behavior contributes directly to reading comprehension and academic success. The Motivational and Engagement Pathway (*n* = 5) underscores intrinsic motivation, persistence, and task engagement as essential mediators through which SEL enhances reading outcomes. The Social Interaction and Prosocial Behavior Pathway (*n* = 7) captures studies focusing on empathy, cooperation, and collaborative meaning-making in classroom literacy contexts. Finally, the Cognitive and Metacognitive Pathway (*n* = 8) integrates findings from large-scale and cross-cultural research showing that SEL improves executive functioning, self-monitoring, and metacognitive awareness, thereby supporting higher-order comprehension skills. This classification not only demonstrates the multifaceted ways SEL influences reading performance but also provides a conceptual foundation for subsequent thematic synthesis and theoretical interpretation.

This section presents findings relevant to the two research questions. The first investigates the impact of SEL on students’ reading achievement. The second explores how teachers can design and implement SEL-integrated instructional materials to boost students’ reading achievement. Findings are drawn from empirical studies, intervention programs, and classroom-based applications.

### 3.1 Q1: How does SEL specifically influence students’ reading achievement?

Many studies show that SEL promotes reading achievement through enhanced emotional regulation. By enabling students to maintain focus, stay calm, and process information more effectively, SEL positively impacts reading performance. For instance, Brackett et al. (2012) found that students in the RULER program showed better decision-making abilities. Similarly, Schonfeld et al. (2015) also reported that students receiving SEL instruction were 1.72 times more likely to achieve basic reading proficiency compared to the control group. These results underscore the crucial role of emotional regulation in promoting reading achievement.

SEL also bolsters students’ intrinsic motivation. Students with enhanced emotional regulation tend to be more curious and resilient. For example, Corcoran et al. (2018) discovered that SEL-enriched classroom settings fostered learning resilience. Panayiotou et al. (2019) similarly found that students with stronger emotional skills showed greater engagement when reading emotionally nuanced narratives. Research by Getty et al. (2021) and Ha and Roehrig (2022) showed that SEL-supported instruction improved reading accuracy, vocabulary acquisition, and task completion rates. These findings emphasize how SEL’s motivational benefits cultivate persistent readers.

Furthermore, SEL enhances classroom social dynamics. Programs on emotional expression, perspective-taking, and peer dialogue establish emotionally safe environments where students feel comfortable sharing interpretations. Ashdown and Bernard (2012) discovered that SEL promoted classroom readiness by encouraging collaborative reading behaviors. Brackett et al. (2012) also reported that group-based emotional reflection deepened textual understanding through shared meaning-making. McCormick et al. (2015) illuminated that students who formed emotional connections with literary characters developed stronger narrative comprehension. These results indicate that SEL establishes socially supportive learning environments that boost reading achievement.

Significantly, the influence of SEL on reading achievement extends beyond short-term benefits. Longitudinal studies indicate that SEL is important in cultivating learning resilience, thereby supporting sustained reading achievement. For instance, McCormick et al. (2015) discovered that early SEL exposure was linked to long-term enhancements in reading. Similarly, Schonfeld et al. (2015) also reported that SEL nurtured transferable skills like goal-setting, persistence, and help-seeking. These long-term effects indicate that SEL provides a robust foundation for lifelong reading achievement.

Collectively, SEL advances reading achievement through multiple, interconnected channels: it enhances emotional regulation, motivation, peer-supported meaning-making, and learning behaviors. SEL should not be viewed as a supplementary teaching approach but as a fundamental component of reading instruction.

### 3.2 Q2: How can teachers design and implement SEL-integrated instructional materials?

Integrating SEL into reading instruction begins with alignment of emotional and academic objectives. Research shows that incorporating emotional vocabulary, empathy-building, and reflective inquiry into literary analysis offers students both cognitive and emotional insight. For example, Kozak and Recchia (2018) demonstrated that reading fiction enhanced students’ social understanding and empathy, especially when they engaged with morally and emotionally complex characters. Similarly, Daunic et al. (2021) developed the SELF curriculum which integrated SEL objectives into story-based literacy lessons, resulting in significant improvements in reading skills and emotional regulation.

Furthermore, learning strategies effectively reinforce the integration of SEL-literacy. Instructional methods like role-playing, storytelling, and journaling enable students to apply emotional understanding while enhancing reading skills. In the PATHS program (Nix et al., 2013), students participated in weekly tasks, such as taking on a character’s perspective or enacting emotionally intense situations which deepened both their reading engagement and socio-emotional competence.

The broader classroom environment is also crucial for SEL-informed reading instruction. Emotionally responsive settings impact reading fluency and comprehension outcomes (Rimm-Kaufman et al., 2007). However, these benefits require culturally responsive and developmentally appropriate implementation. As Jennings and Greenberg (2009) and Wanless and Domitrovich (2015) emphasized, SEL strategies must be tailored to match students’ linguistic, cultural, and socio-emotional backgrounds.

Assessment is integral to the successful integration of SEL into reading instruction. Teachers require practical tools to measure both students’ emotional and academic growth. Daunic et al. (2021) used self-reflection journals and observational checklists to monitor emotional development. Similarly, Pellitteri et al. (2006) showed that incorporating emotional intelligence training into reading instruction enhanced outcomes for students with reading difficulties. Although standardized tools like the Behavior Assessment System for Children (BASC) provide formal means of tracking emotional adjustment, SEL assessments are limited and require further development.

SEL implementation still faces significant challenges. Teachers frequently cite insufficient training, lack of preparation time, and limited access to SEL-aligned instructional materials. Therefore, Corcoran et al. (2018) proposed integrating SEL training into pre-service teacher education programs to enhance educators’ capacity. Mahoney et al. (2018) noted that “studies of all types of educational interventions tend to find that short-term effects are stronger than longer-term effects” (p.21). This widely observed trend highlights the critical importance of sustained, high-quality SEL implementation. However, in under-resourced educational contexts, structural barriers including limited funding, staffing shortages, and policy misalignment further impede the scalability and sustainability of comprehensive SEL models (Todd et al., 2022).

To surmount these hurdles, education systems need to prioritize comprehensive professional development and resource allocation. Providing teachers with adaptable materials is crucial for SEL integration. Additionally, collaborative partnerships between researchers and practitioners can refine teaching models, ensuring they are contextually appropriate and pedagogically robust.

### 3.3 Enhancing reading through SEL: pathways and challenges

The findings of this paper underscore that SEL is a foundational component of effective reading instruction. Grounded in theoretical frameworks like Vygotsky’s sociocultural theory and Zimmerman’s self-regulated learning model, the data validate a dual-pathway model in which SEL boosts reading achievement both cognitively (through focus, metacognition, and self-regulation) and behaviorally (through motivation, persistence, and collaboration).

Table 2 illustrates how five core SEL components influence specific reading skills through targeted cognitive or behavioral mechanisms. This mapping departs from earlier meta-analyses that assessed SEL’s general academic effects (e.g., Durlak et al., 2011; Corcoran et al., 2018) by highlighting reading-specific impacts. For instance, strategies such as character empathy, emotion labeling, and dramatization directly enhance comprehension and inferencing.

**Table 2:** Mapping SEL components to specific reading skills and mechanisms.

SEL component	Target reading skill	Mechanism of influence	Supporting references
Self-control	Reading fluency	Enhances attention, reduces task-switching	Hart et al. (2020) and McKown et al. (2016)
Emotional regulation	Vocabulary acquisition	Supports perseverance through difficult word decoding	Getty et al. (2021) and Ha and Roehrig (2022)
Empathy	Inferential comprehension	Aids understanding of character motives and emotional cues	McCormick et al. (2015), Panayiotou et al. (2019), and Bennett et al. (2023)
Social awareness	Narrative and perspective-taking	Fosters collaboration and shared textual interpretation	Ashdown and Bernard (2012) and Ng and Sun (2022)
Goal-setting and motivation	Reading comprehension and engagement	Builds persistence, fosters reading autonomy	Schonfeld et al. (2015) and Jiang et al. (2024)

Nevertheless, multiple challenges persist. Firstly, implementing SEL in reading instruction is hindered by insufficient teacher training, limited instructional time, and inadequate curricular support. These factors collectively impede the consistent application of SEL strategies (Lu Chen, 2024; Todd et al., 2022). Without systemic investment and leadership support, even the most promising strategies may fail to deliver results in practice (Pham, 2024).

Secondly, there is a significant shortage of culturally responsive and linguistically inclusive SEL tools specifically designed for reading instruction (Cuocci and Arndt, 2020; Lim et al., 2024). Most studies reviewed are from Western contexts, limiting their applicability to multilingual or resource-constrained environments. Future research should prioritize longitudinal and cross-cultural designs to clarify how SEL influences students’ reading achievement across diverse sociocultural settings (Deng, 2022).

Thirdly, the field suffers from a lack of robust and validated assessments capable of measuring both emotional and literacy improvements concurrently (Nemec and Roffey, 2005; Fisher and Frey, 2019; Alemdar and Anilan, 2020). Although programs like BASC provide behavioral data, few tools monitor how emotional growth directly impacts textual comprehension, vocabulary acquisition, or critical thinking (Griffith et al., 2008; Kalil et al., 2023). This gap undermines efforts to evaluate and scale effective interventions.

Despite these limitations, the empirical evidence solidifies its crucial role in advancing reading achievement. For educators, the implications are clear: integrating SEL into reading instruction not only boosts reading achievement but also cultivates the emotional resilience and classroom engagement vital for long-term learning. For researchers, the challenge lies in developing scalable, adaptable frameworks that target both cognitive and affective aspects of reading. Policymakers, meanwhile, face an urgent requirement to embed SEL in curriculum design and teacher preparation.

Building on these results, the following section discusses how the reviewed evidence contributes to theoretical understanding, methodological development, and practical applications of SEL-integrated literacy instruction.

## 4 Discussion

The synthesis of thirty-one studies demonstrates that Social–Emotional Learning (SEL) exerts a consistent, positive influence on students’ reading achievement. Across contexts and methodologies, SEL contributes to literacy growth through complementary emotional, motivational, and cognitive mechanisms. These findings reinforce the argument that reading is not solely a cognitive act but also an emotional and social process shaped by self-regulation, engagement, and interaction (Mardotilla et al., 2024; Pratheesh and Francis, 2024).

A recurring pattern across the evidence is the role of emotional regulation as a foundation for literacy learning. Students who can manage frustration, maintain focus, and control anxiety perform better on comprehension and vocabulary tasks (Brackett et al., 2012; Schonfeld et al., 2015). Emotional competence allows learners to stay engaged during difficult reading experiences, providing the affective stability needed for sustained learning (Bailey et al., 2023). Alongside regulation, intrinsic motivation and curiosity help students persist when faced with challenging texts. Emotionally supportive classrooms stimulate engagement, transforming reading from an obligatory task into a personally meaningful activity (Corcoran et al., 2018; Panayiotou et al., 2019).

SEL also strengthens the social dimension of reading. When classrooms emphasize empathy, dialogue, and perspective-taking, students engage in richer collaborative interpretation (Ashdown and Bernard, 2012; McCormick et al., 2015). Reading becomes a shared practice of constructing meaning rather than a solitary cognitive effort. These conditions foster deeper comprehension and a sense of belonging, two factors that sustain long-term motivation and achievement. Over time, SEL-based instruction nurtures transferable skills such as persistence, reflection, and help-seeking that support lifelong literacy (McCormick et al., 2015; Schonfeld et al., 2015; Prater and Young, 2018).

Despite the converging evidence, several methodological and contextual issues limit generalizability. Most studies employed quasi-experimental or cross-sectional designs, and only a small number tracked outcomes longitudinally. This imbalance makes it difficult to determine the durability of SEL effects or to capture reciprocal interactions between emotional and cognitive growth (Domitrovich et al., 2007). The literature is also dominated by Western samples; only a handful of studies from East Asia or multilingual settings examine how cultural norms influence SEL’s relevance and effectiveness (Wong et al., 2023; Lim et al., 2024; Chen, 2024). These gaps suggest that future research should expand to more diverse contexts and use multi-wave designs that explore how affective and literacy skills co-develop.

Another limitation lies in assessment. Existing tools often treat SEL and reading as separate constructs, preventing clear identification of mediating pathways (Griffith et al., 2008; Kalil et al., 2023). The field would benefit from dual-domain instruments capable of measuring emotional growth alongside literacy performance. Similarly, teacher preparedness remains uneven. Effective SEL integration depends on educators who can model emotional awareness and embed it within literacy instruction. Many teachers, however, lack systematic training or the curricular flexibility needed to sustain these practices (Daunic et al., 2021; Rimm-Kaufman et al., 2007; Lustick and Ota, 2021).

The practical implications are significant. Embedding SEL into literacy education should not be viewed as an add-on but as a structural component of teaching and learning. Classrooms that combine emotional safety with intellectual challenge help students regulate effort, interpret texts empathetically, and engage deeply with meaning. For teacher education, integrating SEL into pre-service and in-service training can ensure that educators possess the emotional literacy required to foster such environments (Corcoran et al., 2018; Sugishita, 2019; Zilva, 2023; Todd et al., 2022). Institutional support—including curriculum design, policy alignment, and professional development—is essential to maintain fidelity and scalability.

At a broader level, the findings have policy relevance. Educational systems should institutionalize SEL within national literacy frameworks, ensuring that reading curricula include emotional and interpersonal learning goals. Assessment standards must evolve to capture the joint development of academic and social–emotional competencies. Funding mechanisms that encourage longitudinal and cross-cultural studies would strengthen the evidence base and guide culturally responsive adaptations.

Overall, this review positions SEL as a transformative framework for literacy instruction. By uniting cognitive, emotional, and social dimensions, SEL reframes reading as a holistic learning process that develops both intellect and empathy. Classrooms that embrace SEL principles cultivate students who can interpret complex texts and navigate human experience with reflection and compassion. Such integration aligns academic excellence with the broader educational goal of nurturing thoughtful, emotionally balanced, and socially responsible learners.

## 5 Conclusion

This review delves into how SEL contributes to students’ reading achievement and how SEL can be effectively incorporated into reading instruction. By synthesizing evidence from 31 articles, the results indicate that SEL boosts reading achievement via four key mechanisms: enhancing emotional regulation, nurturing intrinsic motivation, facilitating peer-supported meaning-making construction, and cultivating long-term academic habits. These mechanisms enable students to engage profoundly with texts by establishing emotional connection and collaborative interpretation. SEL does not simply accompany cognitive development; it constitutes the very context within which higher-order literacy skills emerge. By conceptualizing reading as both an intellectual and an emotional process, this review bridges two research traditions that have historically evolved in isolation, academic achievement and social–emotional development.

The practical implications of these insights are profound. Effective literacy instruction requires a deliberate alignment between emotional and academic goals. Teachers who integrate SEL strategies such as empathy-based discussion, reflective journaling, or dramatization of texts cultivate classrooms where emotional safety and cognitive challenge coexist. Such environments encourage students to explore meaning, manage frustration, and engage deeply with texts. However, this potential can only be realized when educators themselves are equipped with the competencies to model emotional literacy. Teacher training therefore stands as the pivotal link between theory and practice. Integrating SEL into pre-service and in-service programs can empower educators to design lessons that are simultaneously intellectually demanding and emotionally supportive, ensuring that reading instruction nurtures both comprehension and compassion.

Beyond the classroom, the review also underscores the necessity for systemic reform. Education policies should institutionalize SEL within national literacy curricula, framing it as a fundamental component of academic excellence rather than an auxiliary enrichment. Assessment systems must evolve to capture the interplay between emotional development and reading achievement, adopting tools that measure these domains concurrently. Furthermore, research funding and policy incentives should support longitudinal and cross-cultural studies to determine how SEL operates in diverse linguistic and cultural contexts. Only through such institutional commitment can SEL move from a series of promising interventions to a sustainable foundation for equitable literacy education. Nonetheless, the current evidence base remains geographically concentrated and methodologically uneven, underscoring the need for longitudinal and cross-cultural validation of SEL’s effects on literacy.

The global relevance of these findings is particularly salient in the current educational climate. As classrooms become increasingly diverse and digitally mediated, the ability to read with empathy, emotional discernment, and critical reflection has never been more essential. SEL offers a framework for cultivating these competencies, enabling students to navigate not only textual complexity but also the emotional and ethical dimensions of contemporary communication. In this sense, SEL-integrated literacy education responds to the broader mission of schooling—to prepare learners who can think critically, feel deeply, and act responsibly.

Subsequently, this review emphasizes the necessity of redefining SEL from an adjunct program to a fundamental aspect of reading pedagogy. For teachers, this entails creating learning environments and instructional materials that foster emotional skills and reading instruction. For educational policymakers, it requires investment in professional development, curriculum alignment, and research infrastructure. For researchers, it presents new directions for investigating reciprocal relation between students’ emotional competencies and their reading performance.

Ultimately, the cultivation of students’ emotional and cognitive abilities is a potent strategy for enhancing both literacy and overall well-being. As the educational environment continues to evolve, integrating SEL into reading instruction is not merely a pedagogical opportunity but a moral imperative to foster the student comprehensive development.

## Data Availability

The original contributions presented in the study are included in the article/supplementary material, further inquiries can be directed to the corresponding author/s.
